# RANKL–RANK signaling promotes cigarette smoke–induced emphysema with MMP-9 upregulation in alveolar macrophages

**DOI:** 10.3389/fphar.2026.1754836

**Published:** 2026-06-15

**Authors:** Lu Zhou, Baiquan Zhang, Huanqin Wang, Fengxiang Huang, Lijun Miao

**Affiliations:** Department of Respiratory and Critical Care Medicine, The First Affiliated Hospital of Zhengzhou University, Zhengzhou, Henan, China

**Keywords:** alveolar macrophages, COPD, emphysema, MMP-9, RANKL

## Abstract

**Background:**

Chronic obstructive pulmonary disease (COPD) is a leading global health problem, with pulmonary emphysema as one of its hallmark pathological features. Matrix metalloproteinase-9 (MMP-9), predominantly derived from alveolar macrophages, has been implicated in extracellular matrix degradation. However, the upstream regulatory signals responsible for MMP-9 induction in cigarette smoke (CS)–related COPD remain incompletely understood. We investigated whether receptor activator of nuclear factor-κB ligand (RANKL) and its receptor RANK are involved in this process.

**Methods:**

We localized RANKL and RANK in lung tissues of mice subjected to long-term CS exposure. Emphysema was evaluated in CS-exposed mice that received intraperitoneal injections of either an anti-mouse RANKL monoclonal antibody or a rat IgG2a kappa isotype control antibody. Next, we examined their expression under cigarette smoke extract (CSE) stimulation in the MH-S mouse alveolar macrophage cell line. Finally, we evaluated the functional role of RANKL in regulating CS-induced MMP-9 production using neutralizing antibodies.

**Results:**

*In vivo*, chronic CS exposure resulted in alveolar enlargement, structural destruction, and decline in lung function, accompanied by increased expression of RANKL, RANK, and MMP-9 in lung tissue. These molecules were predominantly localized to alveolar macrophages. Neutralization of RANKL was associated with reduced MMP-9 expression, attenuated alveolar damage, and improved pulmonary function. *In vitro*, CSE stimulation of MH-S cells upregulated RANKL and RANK and induced MMP-9 expression, while RANKL blockade partially inhibited this effect.

**Conclusion:**

RANKL–RANK signaling is associated with increased MMP-9 expression in alveolar macrophages and contributes to CS-induced emphysema. Targeting this pathway attenuates structural damage and functional impairment and may represent a potential therapeutic strategy in COPD-related emphysema.

## Introduction

Chronic obstructive pulmonary disease (COPD) is a leading cause of morbidity and mortality worldwide, accounting for approximately three million deaths annually and affecting over 10% of the global population (Boers et al., 2023). Characterized by persistent and largely irreversible airflow limitation, COPD is primarily driven by chronic exposure to noxious particles or gases, with cigarette smoke being the most prominent risk factor (Adeloye et al., 2022; Lei et al., 2024). Among the major pathological subtypes of COPD, pulmonary emphysema is defined by alveolar wall destruction, loss of elastic recoil, and impaired gas exchange (Mocchegiani et al., 2011).

In addition to its local pulmonary effects, cigarette smoke exposure contributes to systemic manifestations of COPD, including osteoporosis. Previous studies have reported elevated circulating levels of receptor activator of nuclear factor-κB ligand (RANKL) in COPD patients with concurrent osteoporosis, suggesting a role for RANKL in COPD pathogenesis beyond the skeletal system (Bai et al., 2011). RANKL, a member of the tumor necrosis factor (TNF) superfamily, binds to its receptor RANK and activates downstream NF-κB signaling pathways involved in inflammation and tissue remodeling (Theoleyre et al., 2004).

Matrix metalloproteinase-9 (MMP-9) is a proteolytic enzyme that degrades extracellular matrix components such as elastin and has been implicated in alveolar wall degradation and the development of emphysema (Elkington and Friedland, 2006). Predominantly produced by alveolar macrophages, MMP-9 is significantly upregulated in the lungs of individuals with smoking-related COPD compared to both healthy smokers and non-smokers (Kang et al., 2003). Cigarette smoke has been shown to induce MMP-9 expression via NF-κB pathway activation in macrophages (Zhou et al., 2019; Kim et al., 2009). However, airway epithelial cells may engage alternative mechanisms such as WNT signaling (Royer et al., 2017; Heijink et al., 2016). In transgenic mouse models, lung-specific overexpression of MMP-9 leads to marked alveolar destruction and emphysematous remodeling, supporting a role for MMP-9 in smoke-induced lung injury (Foronjy et al., 2008; Anderson et al., 1997).

Despite increasing recognition of MMP-9 in COPD, the upstream regulatory signals controlling its expression in alveolar macrophages during chronic cigarette smoke exposure remain incompletely understood. Previous studies, including our own work, have shown that RANKL signaling can regulate MMP-9 expression in alveolar macrophages, particularly under *in vitro* conditions. However, the functional relevance of this pathway *in vivo*, especially in the context of cigarette smoke–induced emphysema, remains to be established. In this study, we aimed to evaluate the role of RANKL/RANK signaling in cigarette smoke–induced emphysema using a murine model of chronic cigarette smoke exposure, combined with an *in vitro* alveolar macrophage model stimulated with cigarette smoke extract (CSE). We further assessed the effects of RANKL neutralization on emphysematous pathology, lung function, and MMP-9 expression. A better understanding of this pathway may provide insights into its functional significance and potential as a therapeutic target in COPD.

## Materials and methods

### Animals and CS exposure

Wild-type C57BL/6 mice (6–8 weeks old) were housed in a specific pathogen-free (SPF) facility with a 12-h light/dark cycle, ambient temperature maintained at 24 °C ± 2 °C, and free access to sterilized food and water. Mice were randomly divided into four groups (n = 8 per group): an air-exposed control group, a CS exposure group, a CS exposure group treated with anti-RANKL monoclonal antibody, and a CS exposure group treated with an isotype-matched control antibody (rat IgG2a, κ). CS exposure was performed using a whole-body smoke exposure system. Mice in the CS-exposed groups were subjected to mainstream cigarette smoke generated from filtered Hongqiqu cigarettes (Henan, China; 11 mg tar, 0.9 mg nicotine, and 11 mg carbon monoxide per cigarette), for two 60-min inhalation sessions per day (with a 20-min interval), 6 days per week, for a total of 24 weeks. Control mice were exposed to filtered room air under the same conditions. Mice in the CS + anti-RANKL group and CS + isotype control group received intraperitoneal injections of 100 μg anti-RANKL monoclonal antibody or isotype-matched control antibody, respectively, 1 h before each CS exposure and administered twice weekly at 3-day intervals. Air-exposed control mice were injected with 0.2 mL phosphate-buffered saline (PBS) on the same schedule.

### Lung function

Following parameter calibration, awake and unrestrained mice were placed in an airtight chamber of a whole-body plethysmography (WBP) system (Buxco FinePointe, Data Sciences International, United States) for measurement. Respiratory data were recorded over 4 min of quiet breathing. The plethysmograph signal was used to measure respiratory parameters, including peak inspiratory flow rate (PIF), peak expiratory flow rate (PEF), mid-expiratory flow rate (EF50), tidal volume (TV), and minute ventilation (MV).

### Lung histology and measurement of emphysema

Mouse lung tissues were harvested, and the lungs were inflated with 4% paraformaldehyde at a constant pressure of 20–25 cmH_2_O via tracheal cannulation prior to fixation. The tissues were then fixed in 4% paraformaldehyde for 24 h, embedded in paraffin, and sectioned at a thickness of 4 μm. Sections were stained with hematoxylin and eosin (H&E) for histological examination.

Pulmonary emphysema was quantified by evaluating alveolar airspace enlargement and alveolar wall destruction. The mean linear intercept (MLI) was measured using a 100 × 100 μm grid randomly superimposed on lung sections. MLI was calculated by dividing the total length of grid lines by the number of alveolar intercepts (Bracke et al., 2006). The destructive index (DI) was assessed using a 42-point grid, with each point falling on alveolar and duct structures classified as normal (N) or destroyed (D). Points overlying other structures (e.g., bronchial walls, blood vessels) were excluded from analysis. DI was calculated as D/(D + N) ×100 (Saetta et al., 1985).

### Immunohistochemistry and immunofluorescence

Lung tissues were collected from mice, fixed in 10% neutral-buffered formalin for 24 h, embedded in paraffin, and sectioned at a thickness of 5 μm. Sections were deparaffinized, rehydrated through graded ethanol, and treated with 0.3% hydrogen peroxide for 15 min to block endogenous peroxidase activity. Antigen retrieval was performed by heating the sections in 5 mM citrate buffer (pH 6.0) in a microwave oven for 15 min. After cooling to room temperature, sections were blocked with goat serum (ZLI-6056; ZSGB-Bio, Beijing, China) for 30 min.

For immunohistochemistry (IHC), sections were incubated overnight at 4 °C with primary antibodies against RANKL (Abcam, Cambridge, United Kingdom), RANK (Abcam), or MMP-9 (Abcam). After washing with Tris-buffered saline (TBS), sections were incubated with an HRP-conjugated goat anti-mouse IgG secondary antibody (PV-6002; ZSGB-Bio) for 30 min at room temperature. Immunoreactivity was visualized using DAB substrate solution (ZLI-9018; ZSGB-Bio), and nuclei were counterstained with hematoxylin. Slides were dehydrated, cleared, and mounted for observation under a light microscope. Negative controls were processed in parallel with omission of the primary antibody.

For immunofluorescence (IF), sections were incubated overnight at 4 °C with primary antibodies including anti-RANKL (Abcam), anti-F4/80 (Abcam), anti-RANK (Abcam), or anti-MMP-9 (Abcam). After PBS washing, sections were incubated with fluorophore-conjugated secondary antibodies—Alexa Fluor® 488 goat anti-rabbit IgG and Alexa Fluor® 594 goat anti-mouse IgG (Jackson ImmunoResearch, West Grove, PA, United States)—for 30 min at 37 °C. Nuclei were counterstained with DAPI. Sections were mounted using antifade medium and stored at 4 °C. Fluorescent signals were visualized using a laser scanning confocal microscope (TCS SP8; Leica Microsystems, Wetzlar, Germany). Negative controls were included by omitting the primary antibodies.

### Cell culture

The MH-S mouse alveolar macrophage cell line was purchased from Wuhan Servicebio Technology Co., Ltd. (Wuhan, Hubei, China). Cells were maintained in Roswell Park Memorial Institute Medium 1640 (A1049101, Thermo Fisher Scientific) supplemented with 10% fetal bovine serum (FBS, 164210, Pricella) and 1% penicillin–streptomycin solution (G4003; Servicebio). Cultures were incubated at 37 °C in a humidified atmosphere containing 5% CO_2_.

### CSE preparation

CSE was prepared by bubbling the smoke of five filtered Baisha cigarettes through 10 mL of RPMI-1640 medium (Thermo Fisher Scientific) at a constant airflow, with each cigarette burned over 5 min. The resulting smoke-conditioned medium was filtered through a 0.22 μm membrane to remove particulate matter, yielding a 100% CSE stock solution. The stock was standardized by adjusting its optical density (OD_320_) to 4.0 ± 0.05 (Wirtz and Schmidt, 1996; Le et al., 2020). Cell proliferation assays (Cell Counting Kit-8) identified 0.5% CSE as the optimal experimental concentration after testing serial dilutions (0.1%, 0.25%, 0.5%, 1%, 2%, 4%).

### Cell stimulation

MH-S alveolar macrophages were cultured at 37 °C in a humidified incubator containing 5% CO_2_ and allocated into five experimental groups: control (standard culture medium), 0.5% CSE treatment, recombinant RANKL stimulation (PeproTech, United States; Cat. No. 315-11), 0.5% CSE combined with anti-RANKL neutralizing antibody (BioLegend, United States; Cat. No. 510010), and 0.5% CSE combined with an isotype-matched control antibody (rat IgG2a, κ). Cells were stimulated with 0.5% CSE for 24 h to assess the expression of RANKL and RANK. Recombinant RANKL, anti-RANKL antibody, and the isotype control antibody were also administered for 24 h to evaluate MMP-9 expression.

### Western blot analysis

Protein expression levels of RANKL, RANK, and MMP-9 were analyzed by Western blotting in both lung tissues and MH-S alveolar macrophages. Lung tissues were homogenized and lysed in ice-cold RIPA buffer containing protease and phosphatase inhibitors (Servicebio, China). MH-S cells were harvested and similarly lysed in RIPA buffer. Protein concentrations were determined using a BCA assay kit (Thermo Fisher Scientific, United States), and equal amounts of protein (30 μg) were resolved on 10% SDS-PAGE gels and transferred to PVDF membranes (Merck-Millipore, Solna, Sweden), which were then blocked and incubated with antibodies: RANK (Abcam), RANKL (Abcam), MMP-9 (Abcam), and β-actin (Abcam). After incubation with HRP-conjugated goat anti-mouse IgG antibody, the immunoreactive bands were detected using the enhanced chemiluminescence from Millipore Company (Bedford, MA). Quantitative image analysis was performed with ImageJ software (NIH, Bethesda, MD). Results are expressed as relative densities.

### Gene expression analysis by Real-Time Quantitative PCR

Real-Time Quantitative PCR (qPCR) was performed to quantify the gene expression levels of MMP-9, RANKL, and RANK in MH-S alveolar macrophages. Total RNA was extracted using an RNA Extraction Kit (AGbio, China) according to the manufacturer’s instructions. Reverse transcription was carried out using the Evo M-MLV RT Kit (AGbio, China). qPCR amplification was performed with the SYBR® Green Premix Pro Taq HS qPCR Kit (AGbio, China) on a QuantStudio 5 Real-Time PCR System (Thermo Fisher Scientific, United States).

The primer sequences were as follows:

GAPDH: 5′-GCA​AAT​TCA​ACG​GCA​CAG​TCA​AG-3′ (forward) and 5′-TCG​CTC​CTG​GAA​GAT​GGT​GAT​G-3′ (reverse);

RANKL: 5′-TAC​CTG​TAC​GCC​AAC​ATT​TGC​TTT​C-3′ (forward) and 5′-TTC​GTG​CTC​CCT​CCT​TTC​ATC​AG-3′ (reverse);

RANK: 5′-ACT​GAG​GAG​ACC​ACC​CAA​GGA​G-3′ (forward) and 5′-GCA​GCC​ACT​ACT​ACC​ACA​GAG​ATG-3′ (reverse);

MMP-9: 5′-CGT​GTC​TGG​AGA​TTC​GAC​TTG​A-3′ (forward) and 5′-TGG​TTC​ACC​TCA​TGG​TCC​AC-3′ (reverse).

Relative gene expression was calculated using the 2^−ΔΔCt^ method, with GAPDH as the internal reference.

### Flow cytometry

At the end of the culture period, MH-S cells from the control and 0.5% CSE–treated groups were harvested from 6-well plates for flow cytometric analysis. Cells were incubated with fluorochrome-conjugated antibodies, including phycoerythrin (PE)–conjugated anti-mouse RANKL (BioLegend, CA, United States) and allophycocyanin (APC)–conjugated anti-mouse RANK (BioLegend), along with appropriate isotype control antibodies (PE-conjugated rat IgG2a κ and APC-conjugated rat IgG2a κ; BioLegend). After staining, a minimum of 10,000 events per sample were acquired on a BD FACSCalibur™ flow cytometer and analyzed using FlowJo software (Tree Star Inc., Ashland, OR, United States).

### Statistical analysis

All experiments were repeated at least three times, and the data were presented as the mean ± standard error of the mean (SEM). For normally distributed data, comparisons among three or more groups were conducted using one-way ANOVA. For non-normally distributed data, multiple-group comparisons were analyzed using Tamhane’s T2 test. Comparisons between two groups were performed using Student’s t-test. A *P*-value <0.05 was considered statistically significant. Statistical analyses were performed using GraphPad Prism software (version 9.0; GraphPad Software, San Diego, CA, United States).

## Results

### RANKL and RANK expression are elevated in lung tissues following CS exposure

**FIGURE 1:**
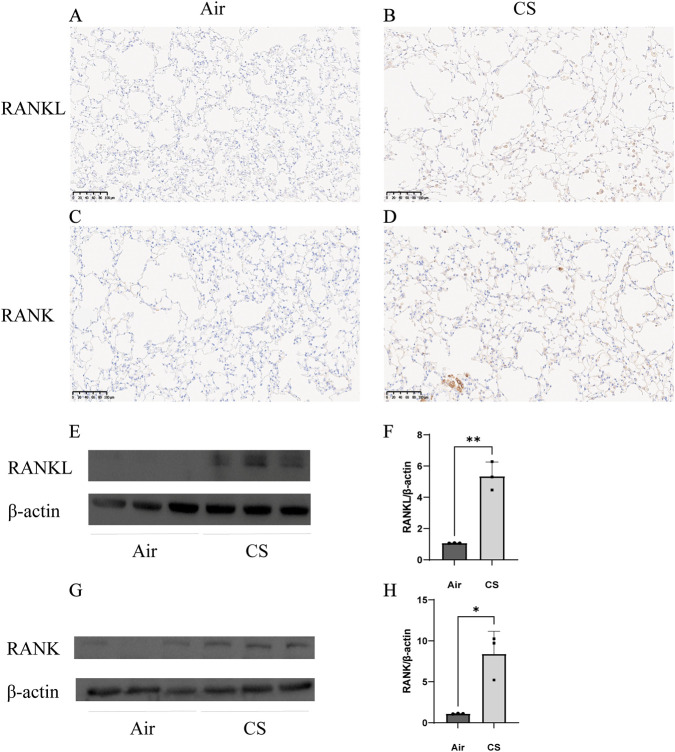
Detection of RANKL and RANK expression in lung tissues. RANKL expression was detected in air-exposed **(A)** and CS-exposed **(B)** mice; RANK expression was detected in air-exposed **(C)** and CS-exposed **(D)** mice. Immunoreactivity is indicated by brown chromogenic staining using DAB. CS exposure markedly upregulated RANKL and RANK expression, predominantly in the alveolar regions. Scale bar = 100 μm. Western blot analysis of RANKL **(E,F)** and RANK **(G,H)** protein expression in lung tissues. Data are presented as mean ± SEM (n = 3 per group). *P < 0.05, **P < 0.01. Abbreviations: CS, cigarette smoke; DAB, 3,3′-diaminobenzidine.

After 24 weeks of chronic cigarette smoke (CS) exposure, immunohistochemical analysis revealed markedly increased expression of RANKL (Figure 1B) and RANK (Figure 1D) in the lungs of CS-exposed mice, particularly within the alveolar regions. In contrast, RANKL (Figure 1A) and RANK (Figure 1C) expression was barely detectable in air-exposed controls. Western blot analysis further confirmed elevated protein levels of RANKL (Figures 1E,F) and RANK (Figures 1G,H) following CS exposure.

### Chronic CS exposure induces emphysematous changes and impaired lung function, ameliorated by Anti-RANKL treatment

**FIGURE 2:**
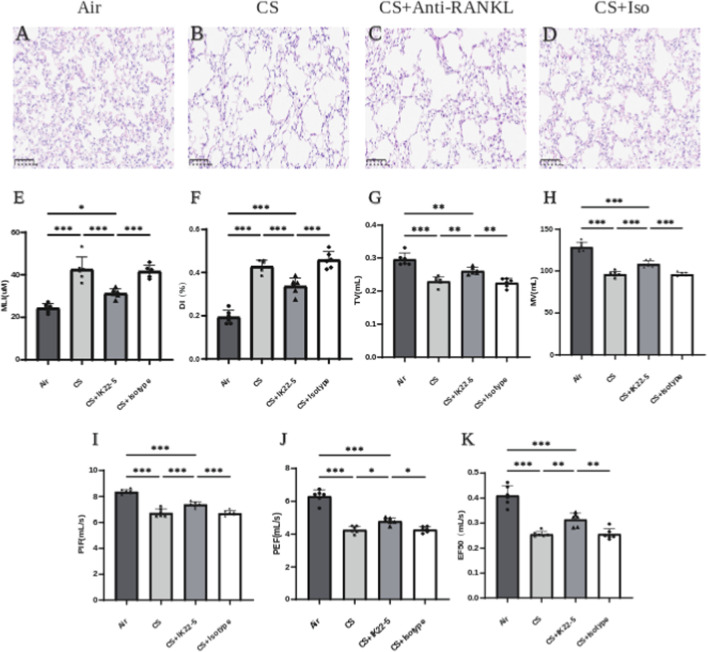
Histological morphology, quantification of alveolar damage, and pulmonary function in mice. **(A–D)** Alveolar regions from mice exposed to air **(A)**, CS **(B)**, CS with anti-RANKL antibody treatment **(C)**, and CS with isotype control antibody **(D)**. All sections were stained with H&E (400×). Scale bar = 100 μm. **(E,F)** Morphometric analysis of lung sections showing mean linear intercept (MLI) **(E)** and destructive index (DI) **(F)** in each group. **(G–K)** Pulmonary function parameters including tidal volume (TV) **(G)**, minute ventilation (MV) **(H)**, peak inspiratory flow (PIF) **(I)**, peak expiratory flow (PEF) **(J)**, and expiratory flow at 50% tidal volume (EF50) **(K)**. Data are presented as mean ± SEM (n = 6 per group). *P < 0.05, **P < 0.01, ***P < 0.001. Statistical analysis was performed using one-way ANOVA with *post hoc* testing. Abbreviations: CS, cigarette smoke; H&E, hematoxylin and eosin; MLI, mean linear intercept; DI, destructive index; TV, tidal volume; MV, minute ventilation; PIF, peak inspiratory flow; PEF, peak expiratory flow; EF50, expiratory flow at 50% tidal volume; SEM, standard error of the mean.

Significant pathological alterations in lung architecture and function were observed in CS-exposed mice. Compared with air-exposed controls, CS exposure resulted in enlarged alveolar spaces, thinning of alveolar septa, and destruction of alveolar walls (Figures 2A,B). Anti-RANKL treatment markedly attenuated these structural changes compared with both the CS-exposed group and the isotype antibody control group (Figures 2C,D). Quantitative analysis revealed significant increases in mean linear intercept (MLI) and alveolar destructive index (DI) in CS-exposed mice compared to air controls. Anti-RANKL administration significantly reduced both MLI and DI values compared to the CS and isotype antibody groups (Figures 2E,F). Pulmonary function tests demonstrated impairment in CS-exposed mice, with decreases in tidal volume (TV) (Figure 2G), minute ventilation (MV) (Figure 2H), peak inspiratory flow (PIF) (Figure 2I), peak expiratory flow (PEF) (Figure 2J), and expiratory flow at 50% tidal volume (EF50) (Figure 2K) compared to air controls. Anti-RANKL treatment partially restored these parameters (Figures 2G–K).

### CS exposure upregulates MMP-9 expression, which is attenuated by Anti-RANKL treatment

**FIGURE 3:**
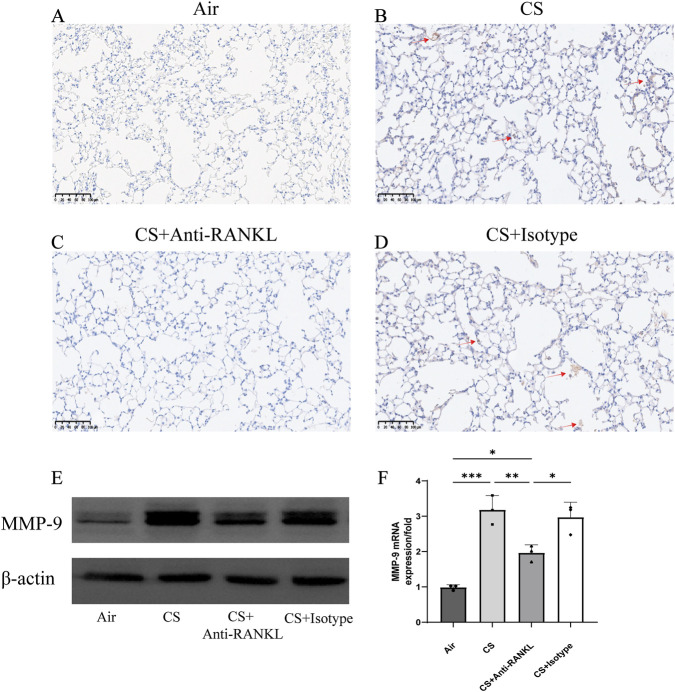
Detection of MMP-9 expression in lung tissues. Immunohistochemical staining of MMP-9 expression in the lungs of mice from the Air **(A)**, CS **(B)**, CS + anti-RANKL **(C)**, and CS + isotype control **(D)** groups. Positive immunoreactivity is indicated by brown DAB staining. Western blot analysis was used to assess MMP-9 protein levels in lung tissues **(E)**. qPCR was performed to quantify MMP-9 mRNA expression **(F)**. *P < 0.05, **P < 0.01, ***P < 0.001. Abbreviations: CS, cigarette smoke; DAB, 3,3′-diaminobenzidine; SEM, standard error of the mean.

Immunohistochemical staining showed that, compared with air-exposed mice (Figure 3A), MMP-9 expression was markedly increased in the lung tissues of CS-exposed mice (Figure 3B). Anti-RANKL treatment (Figure 3C) reduced MMP-9 immunoreactivity relative to both the CS group (Figure 3B) and the isotype control group (Figure 3D). Western blot analysis (Figure 3E) confirmed that CS exposure increased MMP-9 protein expression compared with air controls, and anti-RANKL treatment reduced this upregulation. In addition, qPCR analysis (Figure 3F) demonstrated a parallel increase in MMP-9 mRNA expression, which was partially reversed by anti-RANKL administration.

### RANKL, RANK, and MMP-9 are predominantly expressed in alveolar macrophage

**FIGURE 4:**
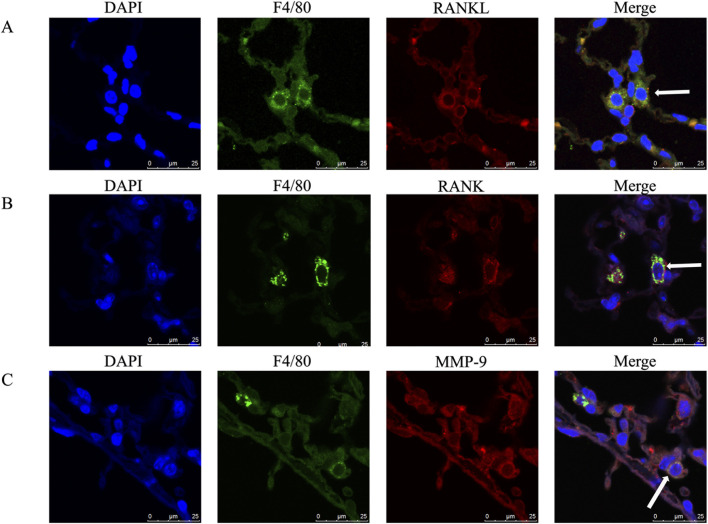
Cellular localization of RANKL, RANK, and MMP-9 in lung tissues of CS-exposed mice. **(A)** Immunofluorescence staining showing RANKL (red, Alexa Fluor 594) co-localized with F4/80 (green, Alexa Fluor 488) in lung sections. **(B)** Immunofluorescence staining of RANK (red) and F4/80 (green). **(C)** Immunofluorescence staining of MMP-9 (red) and F4/80 (green). All sections were counterstained with DAPI (blue). Co-expressing F4/80^+^ cells are indicated by white arrows. Scale bar = 25 μm.

2Immunofluorescence analysis of lung sections from CS-exposed mice showed that RANKL, RANK, and MMP-9 were primarily localized to F4/80^+^ alveolar macrophages. Co-localization with F4/80 and nuclear staining (DAPI) was observed in the alveolar compartments (Figures 4A–C), indicating that alveolar macrophages are a major source of these molecules.

### RANKL/RANK axis mediates CSE-Induced MMP-9 expression in alveolar macrophages

**FIGURE 5:**
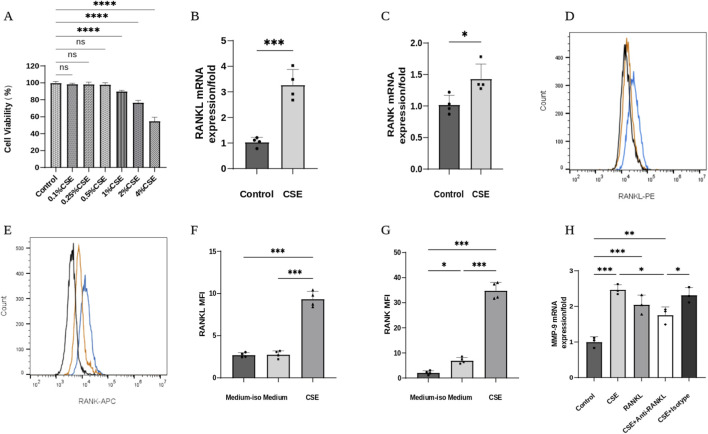
CSE stimulation upregulates RANKL, RANK, and MMP-9 expression in MH-S alveolar macrophages *in vitro* **(A)** CCK-8 assay was used to determine the optimal concentration of CSE for MH-S cell stimulation, with 0.5% CSE selected as the working concentration. **(B,C)** qPCR analysis showing that 0.5% CSE stimulation significantly increased mRNA expression of RANKL **(B)** and RANK **(C)** compared with control cells. **(D–G)** Flow cytometry analysis of surface expression of RANKL **(D,F)** and RANK **(E,G)** in MH-S cells. Exposure to 0.5% CSE significantly increased MFI compared with controls. Black: Medium + isotype control; Brown: Medium alone; Blue: 0.5% CSE stimulation. **(H)** qPCR analysis showing that MMP-9 mRNA expression was also elevated following 0.5% CSE exposure, and this induction was partially suppressed by anti-RANKL antibody compared with the CSE and isotype control groups. Data are presented as mean ± SEM. *P < 0.05, **P < 0.01, ***P < 0.001. Abbreviations: CSE, cigarette smoke extract; MFI, mean fluorescence intensity; qPCR, quantitative polymerase chain reaction; SEM, standard error of the mean.

A CCK-8 assay was first performed to determine the optimal concentration of CSE for MH-S cell stimulation. A concentration of 0.5% CSE was ultimately selected as the working dose (Figure 5A). qPCR analysis showed that 0.5% CSE stimulation significantly increased RANKL (Figure 5B) and RANK (Figure 5C) mRNA expression in MH-S alveolar macrophages compared with untreated controls. Flow cytometric analysis confirmed that surface expression levels of RANKL (Figures 5D,F) and RANK (Figures 5E,G) were elevated in CSE-treated MH-S cells compared with unstimulated controls. In addition, CSE treatment markedly upregulated MMP-9 mRNA expression, which was partially inhibited by co-treatment with an anti-RANKL antibody, with significant differences compared with both the CSE group and the isotype control group (Figure 5H).

## Discussion

In this study, we demonstrate that upregulation of MMP-9 in alveolar macrophages is associated with activation of the RANKL/RANK signaling axis in the context of CS–induced emphysema. This conclusion is supported by both a murine model of chronic CS exposure and *in vitro* experiments using CSE-stimulated macrophages. Chronic CS exposure significantly increased the expression of RANKL, RANK, and MMP-9 in lung tissues, and immunofluorescence confirmed their predominant localization in alveolar macrophages. Importantly, functional inhibition of RANKL reduced MMP-9 expression and was accompanied by attenuation of alveolar destruction and improvement in multiple lung function parameters. To our knowledge, this study provides direct *in vivo* functional evidence that targeting RANKL attenuates emphysematous pathology and improves lung function.

Beyond its local pulmonary effects, the RANKL/RANK axis may also contribute to systemic manifestations of COPD (Song et al., 2022). Osteoporosis is a common comorbidity in COPD, and both bone mineral density loss and fracture risk correlate with disease severity and emphysema phenotypes (Chen et al., 2016). Clinical studies have reported disturbances of the OPG/RANK/RANKL pathway in COPD patients, particularly in those with systemic inflammation and emphysema, raising the possibility that RANKL functions as an “osteokine” linking bone and lung pathology (Mou et al., 2024; Kovács et al., 2019). As members of the TNF receptor superfamily, RANKL and its receptor RANK are known to regulate bone remodeling, lymphoid tissue development, and immune responses, and are expressed in multiple tissues including the thymus, bone, mammary gland, and lung (Walsh and Choi, 2014; Ono et al., 2020; Xiong et al., 2021). In COPD patients and in CS-exposed murine models, elevated RANKL and RANK expression has been observed in lung tissue and lymphoid follicles, with levels correlating with smoking history and lung function decline (Xiong et al., 2020; Zhou et al., 2019). Beyond COPD, RANKL has also been implicated in granuloma formation in tuberculosis, fibroblast activation in pulmonary fibrosis, and modulation of the tumor immune microenvironment in lung cancer, highlighting its broad involvement in pulmonary immune regulation and structural remodeling (van Dam et al., 2019). Nevertheless, the specific pathological contribution of RANKL/RANK signaling to CS-induced alveolar injury has remained unclear, and our study demonstrates that RANKL neutralization attenuates emphysematous pathology and is associated with reduced MMP-9 expression, thereby supporting a role for RANKL signaling in emphysema pathogenesis.

MMP-9, a zinc-dependent endopeptidase, degrades diverse ECM components such as collagen, gelatin, and elastin (Atkinson et al., 2011). While MMP-9 contributes to physiological ECM turnover and repair, its pathological overactivation leads to destruction of the alveolar basement membrane and elastic fibers, hallmarks of emphysematous remodeling (Vu and Werb, 2000; Russell et al., 2002). Clinical studies consistently report elevated levels of MMP-9 in sputum and lung tissue of smokers and COPD patients, with levels correlating with lung function decline, airway remodeling, frequency of acute exacerbations, and mortality risk (Ilumets et al., 2007; Kang et al., 2003; Koo et al., 2016). Thus, MMP-9 is considered not only a contributor to ECM remodeling but also a potential biomarker of disease progression and prognosis. Our data are consistent with these observations, showing that CS exposure upregulates RANKL and RANK and is accompanied by increased MMP-9 expression in lung tissue. *In vitro* experiments further showed that CSE stimulation and recombinant RANKL increased MMP-9 mRNA expression in macrophages, while RANKL neutralization partially reduced this induction. These findings suggest that RANKL signaling may regulate MMP-9 expression in alveolar macrophages. Taken together, our study extends previous observations by linking RANKL signaling to MMP-9 expression in the context of CS-induced emphysema, while further studies are required to elucidate the underlying mechanisms.

Consistent with previous reports, we observed that RANKL, RANK, and MMP-9 were predominantly localized to F4/80^+^ alveolar macrophages. However, while neutrophils and epithelial cells are also capable of producing RANKL under inflammatory conditions (Hu et al., 2017; Kimura et al., 2019), our findings suggest that alveolar macrophages are the major cellular component responding to CS-induced RANKL, associated with increased MMP-9 expression and emphysematous remodeling. The potential involvement of other cell types, such as neutrophils and epithelial cells, in their expression remains to be investigated further. This observation is further supported by clinical evidence showing elevated circulating RANKL levels in COPD patients with osteoporosis, suggesting that this pathway may serve as a bridge linking systemic immune dysregulation with local pulmonary remodeling (Bai et al., 2011; Xiong et al., 2020).

Several limitations should be acknowledged. First, the causal role of RANKL in alveolar destruction was primarily inferred from functional neutralization. Although effective, genetic confirmation using conditional RANKL deletion models would provide stronger evidence. Global RANKL deficiency results in osteopetrosis, marrow cavity loss, and severe skeletal growth defects, precluding long-term CS exposure studies in such animals. Future work using cell type–specific knockouts may help overcome this limitation (Kong et al., 1999). Second, while alveolar macrophages were the focus of this study, other lung cell types—including epithelial and endothelial cells—may also contribute to RANKL-mediated signaling and should be investigated further (Kim et al., 2002; Min et al., 2003; Akiyama et al., 2012). Third, our findings were derived from murine models and immortalized cell lines, which may not fully capture human COPD pathophysiology. Validation using human lung tissue, ideally with single-cell and spatial transcriptomics, will be essential to confirm the translational relevance. Finally, while we discussed broader implications of RANKL in fibrosis, infection, and cancer, these extrapolations should be interpreted as hypotheses requiring further experimental validation rather than established mechanisms. In addition, immunofluorescence analysis in this study was primarily used for cellular localization, and the lack of single-colour controls represents a technical limitation.

A major strength of this study lies in extending previous observations of RANKL/RANK signaling to an *in vivo* model of cigarette smoke–induced emphysema, while providing functional evidence that RANKL blockade attenuates emphysematous changes and improves lung function. Neutralization of RANKL was associated with structural improvement, as reflected by reduced MLI and DI, as well as partial recovery of pulmonary function parameters (TV, MV, PIF, PEF, and EF50). These effects were accompanied by decreased MMP-9 expression in alveolar macrophages, supporting a role for RANKL signaling in modulating proteolytic activity in the context of CS-induced lung injury. Taken together, these findings indicate that the RANKL/RANK pathway contributes to the pathogenesis of emphysema and may represent a potential target for therapeutic intervention.

## Conclusion

In summary, this study identifies RANKL/RANK signaling as a regulator of MMP-9 production in alveolar macrophages during chronic CS exposure, contributing to extracellular matrix remodeling and alveolar destruction. Beyond these observations, we provide *in vivo* evidence that RANKL blockade attenuates emphysema pathology, improves lung function, and reduces MMP-9 expression. These findings highlight the RANKL/RANK–MMP-9 axis as a functionally relevant pathway in COPD and a potential therapeutic target.

## Data Availability

The raw data supporting the conclusions of this article will be made available by the authors, without undue reservation.

## References

[B1] Adeloye D. Song P. Zhu Y. Campbell H. Sheikh A. Rudan I. (2022). Global, regional, and national prevalence of, and risk factors for, chronic obstructive pulmonary disease (COPD) in 2019: a systematic review and modelling analysis. Lancet Respir. Med. 10, 447–458. 10.1016/S2213-2600(21)00511-7 35279265 PMC9050565

[B2] Akiyama T. Shinzawa M. Akiyama N. (2012). RANKL-RANK interaction in immune regulatory systems. World J. Orthop. 3, 142–150. 10.5312/wjo.v3.i9.142 23173110 PMC3502610

[B3] Anderson D. M. Maraskovsky E. Billingsley W. L. Dougall W. C. Tometsko M. E. Roux E. R. (1997). A homologue of the TNF receptor and its ligand enhance T-cell growth and dendritic-cell function. Nature 390, 175–179. 10.1038/36593 9367155

[B4] Atkinson J. J. Lutey B. A. Suzuki Y. Toennies H. M. Kelley D. G. Kobayashi D. K. (2011). The role of matrix metalloproteinase-9 in cigarette smoke-induced emphysema. Am. J. Respir. Crit. Care Med. 183, 876–884. 10.1164/rccm.201005-0718OC 21057003 PMC3086754

[B5] Bai P. Sun Y. Jin J. Hou J. Li R. Zhang Q. (2011). Disturbance of the OPG/RANK/RANKL pathway and systemic inflammation in COPD patients with emphysema and osteoporosis. Respir. Res. 12, 157. 10.1186/1465-9921-12-157 22176920 PMC3260206

[B6] Boers E. Barrett M. Su J. G. Benjafield A. V. Sinha S. Kaye L. (2023). Global burden of chronic obstructive pulmonary disease through 2050. JAMA Netw. Open 6, e2346598. 10.1001/jamanetworkopen.2023.46598 38060225 PMC10704283

[B7] Bracke K. R. D'Hulst A I. Maes T. Moerloose K. B. Demedts I. K. Lebecque S. (2006). Cigarette smoke-induced pulmonary inflammation and emphysema are attenuated in CCR6-deficient mice. J. Immunol. 177, 4350–4359. 10.4049/jimmunol.177.7.4350 16982869

[B8] Chen Y. Bai P. Liu L. Han J. Zeng H. Sun Y. (2016). Increased RANKL expression in peripheral T cells is associated with decreased bone mineral density in patients with COPD. Int. J. Mol. Med. 38, 585–593. 10.3892/ijmm.2016.2629 27279356

[B9] Elkington P. T. Friedland J. S. (2006). Matrix metalloproteinases in destructive pulmonary pathology. Thorax 61, 259–266. 10.1136/thx.2005.051979 16227332 PMC2080735

[B10] Foronjy R. Nkyimbeng T. Wallace A. Thankachen J. Okada Y. Lemaitre V. (2008). Transgenic expression of matrix metalloproteinase-9 causes adult-onset emphysema in mice associated with the loss of alveolar elastin. Am. J. Physiol. Lung Cell Mol. Physiol. 294, L1149–L1157. 10.1152/ajplung.00481.2007 18408070

[B11] Heijink I. H. DE Bruin H. G. Dennebos R. Jonker M. R. Noordhoek J. A. Brandsma C. A. (2016). Cigarette smoke-induced epithelial expression of WNT-5B: implications for COPD. Eur. Respir. J. 48, 504–515. 10.1183/13993003.01541-2015 27126693

[B12] Hu X. Sun Y. Xu W. Lin T. Zeng H. (2017). Expression of RANKL by peripheral neutrophils and its association with bone mineral density in COPD. Respirology 22, 126–132. 10.1111/resp.12878 27552066

[B13] Ilumets H. Rytilä P. Demedts I. Brusselle G. G. SovijäRVI A. MylläRNIEMI M. (2007). Matrix metalloproteinases -8, -9 and -12 in smokers and patients with stage 0 COPD. Int. J. Chron. Obstruct Pulmon Dis. 2, 369–379. 10.2147/COPD.S12159971 18229576 PMC2695187

[B14] Kang M. J. Oh Y. M. Lee J. C. Kim D. G. Park M. J. Lee M. G. (2003). Lung matrix metalloproteinase-9 correlates with cigarette smoking and obstruction of airflow. J. Korean Med. Sci. 18, 821–827. 10.3346/jkms.2003.18.6.821 14676438 PMC3055149

[B15] Kim Y. M. Kim Y. M. Lee Y. M. Kim H. S. Kim J. D. Choi Y. (2002). TNF-Related activation-induced cytokine (TRANCE) induces angiogenesis through the activation of src and phospholipase C (PLC) in human endothelial cells. J. Biol. Chem. 277, 6799–6805. 10.1074/jbc.M109434200 11741951

[B16] Kim S. E. Thanh Thuy T. T. Lee J. H. Ro J. Y. Bae Y. A. Kong Y. (2009). Simvastatin inhibits induction of matrix metalloproteinase-9 in rat alveolar macrophages exposed to cigarette smoke extract. Exp. Mol. Med. 41, 277–287. 10.3858/emm.2009.41.4.031 19299917 PMC2679231

[B17] Kimura S. Mutoh M. Hisamoto M. Saito H. Takahashi S. Asakura T. (2019). Airway M cells arise in the lower airway due to RANKL signaling and reside in the bronchiolar epithelium associated with iBALT in murine models of respiratory disease. Front. Immunol. 10, 1323. 10.3389/fimmu.2019.01323 31244859 PMC6579949

[B18] Kong Y. Y. Yoshida H. Sarosi I. Tan H. L. Timms E. Capparelli C. (1999). OPGL is a key regulator of osteoclastogenesis, lymphocyte development and lymph-node organogenesis. Nature 397, 315–323. 10.1038/16852 9950424

[B19] Koo H. K. Hong Y. Lim M. N. Yim J. J. Kim W. J. (2016). Relationship between plasma matrix metalloproteinase levels, pulmonary function, bronchodilator response, and emphysema severity. Int. J. Chron. Obstruct Pulmon Dis. 11, 1129–1137. 10.2147/COPD.S103281 27313452 PMC4890689

[B20] KováCS B. Vajda E. Nagy E. E. (2019). Regulatory effects and interactions of the wnt and OPG-RANKL-RANK signaling at the bone-cartilage interface in osteoarthritis. Int. J. Mol. Sci. 20, 4653. 10.3390/ijms20184653 31546898 PMC6769977

[B21] Le Y. Cao W. Zhou L. Fan X. Liu Q. Liu F. (2020). Infection of *Mycobacterium tuberculosis* promotes both M1/M2 polarization and MMP production in cigarette smoke-exposed macrophages. Front. Immunol. 11, 1902. 10.3389/fimmu.2020.01902 32973788 PMC7468417

[B22] Lei J. Huang K. Wu S. Xu J. Xu Y. Zhao J. (2024). Heterogeneities and impact profiles of early chronic obstructive pulmonary disease status: findings from the China pulmonary health study. Lancet Reg. Health West Pac 45, 101021. 10.1016/j.lanwpc.2024.101021 38352242 PMC10862401

[B23] Min J.-K. Kim Y.-M. Kim Y.-M. Kim E.-C. Gho Y. S. Kang I.-J. (2003). Vascular endothelial growth factor up-regulates expression of receptor activator of NF-kappa B (RANK) in endothelial cells. Concomitant increase of angiogenic responses to RANK ligand, J. Biol. Chem. 278, 39548–39557. 10.1074/jbc.M300539200 12893832

[B24] Mocchegiani E. Giacconi R. Costarelli L. (2011). Metalloproteases/Anti-Metalloproteases imbalance in chronic obstructive pulmonary disease: genetic factors and treatment implications. Curr. Opin. Pulm. Med. 17 (Suppl. 1), S11–S19. 10.1097/01.mcp.0000410743.98087.12 22209925

[B25] Mou K. Chan S. M. H. Vlahos R. (2024). Musculoskeletal crosstalk in chronic obstructive pulmonary disease and comorbidities: emerging roles and therapeutic potentials. Pharmacol. Ther. 257, 108635. 10.1016/j.pharmthera.2024.108635 38508342

[B26] Ono T. Hayashi M. Sasaki F. Nakashima T. (2020). RANKL biology: bone metabolism, the immune system, and beyond. Inflamm. Regen. 40, 2. 10.1186/s41232-019-0111-3 32047573 PMC7006158

[B27] Royer P. J. Henrio K. Pain M. Loy J. Roux A. Tissot A. (2017). TLR3 promotes MMP-9 production in primary human airway epithelial cells through Wnt/β-catenin signaling. Respir. Res. 18, 208. 10.1186/s12931-017-0690-y 29237464 PMC5729411

[B28] Russell R. E. Culpitt S. V. Dematos C. Donnelly L. Smith M. Wiggins J. (2002). Release and activity of matrix metalloproteinase-9 and tissue inhibitor of metalloproteinase-1 by alveolar macrophages from patients with chronic obstructive pulmonary disease. Am. J. Respir. Cell Mol. Biol. 26, 602–609. 10.1165/ajrcmb.26.5.4685 11970913

[B29] Saetta M. Shiner R. J. Angus G. E. Kim W. D. Wang N. S. King M. (1985). Destructive index: a measurement of lung parenchymal destruction in smokers. Am. Rev. Respir. Dis. 131, 764–769. 10.1164/arrd.1985.131.5.764 4003921

[B30] Song S. Guo Y. Yang Y. Fu D. (2022). Advances in pathogenesis and therapeutic strategies for osteoporosis. Pharmacol. Ther. 237, 108168. 10.1016/j.pharmthera.2022.108168 35283172

[B31] Theoleyre S. Wittrant Y. Tat S. K. Fortun Y. Redini F. Heymann D. (2004). The molecular triad OPG/RANK/RANKL: involvement in the orchestration of pathophysiological bone remodeling. Cytokine Growth Factor Rev. 15, 457–475. 10.1016/j.cytogfr.2004.06.004 15561602

[B32] Van Dam P. A. Verhoeven Y. Jacobs J. Wouters A. Tjalma W. Lardon F. (2019). RANK-RANKL signaling in cancer of the uterine cervix: a review. Int. J. Mol. Sci. 20, 2183. 10.3390/ijms20092183 31052546 PMC6540175

[B33] Vu T. H. Werb Z. (2000). Matrix metalloproteinases: effectors of development and normal physiology. Genes Dev. 14, 2123–2133. 10.1101/gad.815400 10970876

[B34] Walsh M. C. Choi Y. (2014). Biology of the RANKL-RANK-OPG system in immunity, bone, and beyond. Front. Immunol. 5, 511. 10.3389/fimmu.2014.00511 25368616 PMC4202272

[B35] Wirtz H. R. Schmidt M. (1996). Acute influence of cigarette smoke on secretion of pulmonary surfactant in rat alveolar type II cells in culture. Eur. Respir. J. 9, 24–32. 10.1183/09031936.96.09010024 8834329

[B36] Xiong J. Zhou L. Tian J. Yang X. Li Y. Jin R. (2020). Cigarette smoke-induced lymphoid neogenesis in COPD involves IL-17/RANKL pathway. Front. Immunol. 11, 588522. 10.3389/fimmu.2020.588522 33613513 PMC7892459

[B37] Xiong J. Le Y. Rao Y. Zhou L. Hu Y. Guo S. (2021). RANKL mediates muscle atrophy and dysfunction in a cigarette smoke-induced model of chronic obstructive pulmonary disease. Am. J. Respir. Cell Mol. Biol. 64, 617–628. 10.1165/rcmb.2020-0449OC 33689672

[B38] Zhou L. Le Y. Tian J. Yang X. Jin R. Gai X. (2019). Cigarette smoke-induced RANKL expression enhances MMP-9 production by alveolar macrophages. Int. J. Chron. Obstruct Pulmon Dis. 14, 81–91. 10.2147/COPD.S190023 30587964 PMC6304243

